# Integration of renewable energy sources using multiport converters for ultra-fast charging stations for electric vehicles: An overview

**DOI:** 10.1016/j.heliyon.2024.e35782

**Published:** 2024-08-03

**Authors:** Jayaprakash Suvvala, Sathish Kumar K, C. Dhananjayulu, Hossam Kotb, Ali Elrashidi

**Affiliations:** a School of Electrical Engineering, Vellore Institute of Technology, Vellore, Tamil Nadu, India; b Department of Electrical Power and Machines, Faculty of Engineering, Alexandria University, Alexandria, 21544, Egypt; c Electrical Engineering Department, University of Business and Technology, Ar Rawdah, Jeddah, 23435, Saudi Arabia

**Keywords:** Alternate current(AC), Direct current(DC), DC-DC multiport converters, Extreme fast charging stations, Electric vehicles, Power electronic converters, Renewable energy sources (RES), Solid-state transformers

## Abstract

The rise of electric vehicles (EVs) necessitates an efficient charging infrastructure capable of delivering a refueling experience akin to conventional vehicles. Innovations in Extreme Fast Charging (EFC) offer promising solutions in this regard. By harnessing renewable energy sources and employing sophisticated multiport converters, EFC systems can meet the evolving demands of EV refueling. A single-stage topology simplifies the converter design, focusing on efficient DC-AC conversion, vital for feeding solar power into the grid or charging stations. It provides power factor correction, harmonics filtering, and mitigates power quality issues, ensuring stable and efficient operations. Converters with Maximum Power Point Tracking (MPPT) capability facilitate the efficient integration of solar PV systems in charging stations, ensuring maximum solar energy utilization for EV charging. The ability to operate in different modes allows seamless integration with energy storage systems, storing excess solar energy for use during night-time or peak demand periods, enhancing overall efficiency and reliability. Advanced converters support bidirectional energy flow, enabling EV batteries to discharge back to the grid, aiding grid stability and energy management. However, robust control algorithms are needed to handle dynamic conditions like partial shading more effectively. Our review focuses on integrating renewable energy sources with multiport converters, providing insights into a novel EV charging station framework optimized for EFC topology. We highlight the advantages of multiport non-isolated converters over traditional line frequency transformers, particularly in medium voltage scenarios, offering enhanced efficiency and versatility for EFC applications.

## 1 Introduction

Conventional vehicles (CVs) have both instantaneous and long-term detrimental effects on the environment due to their emissions of a wide range of harmful gases, including carbon dioxide, nitrous oxide, sulfur dioxide, hydrocarbons, carbon monoxide, benzene, and formaldehyde. These emissions contribute significantly to greenhouse gas accumulation, ozone layer depletion, and acid rain. They also pose severe health risks, causing skin and eye irritation, allergies, respiratory issues, hearing damage, and psychological health problems. Below, Table 1 presents detailed information comparing electric vehicles (EVs) and conventional vehicles (CVs) across various factors.

**Table 1:** Comparison of EVs vs CV's.

Category	Electric Vehicles (EVs)	Conventional Vehicles (CVs)
**Environmental Impact**	**Advantages(adv):** Lower emissions (zero tailpipe emissions) And Can be charged using renewable energy sources **Disadvantage(Dis):** Environmental impact of battery production and disposal	**Adv**: None **Dis**: High emissions (CO_2_, NOx, SO_2_, hydrocarbons, CO, benzene, formaldehyde) & Dependence on fossil fuels leading to resource depletion and environmental degradation
**Efficiency**	**Adv**: High energy efficiency (over 85 % conversion efficiency) & Regenerative braking **Dis**: Lower energy density of batteries compared to gasoline	**Adv**: High energy density due to gasoline **Dis**: Low energy efficiency (20–30 % conversion efficiency) & No Regenerative braking
**Operational Costs**	**Adv**: Lower fuel costs (electricity cheaper than gasoline) & Reduced maintenance (fewer moving parts) **Dis**: Higher initial purchase price due to battery costs & Battery replacement costs	**Adv**: None **Dis**: Higher fuel costs (gasoline/diesel generally more expensive than electricity) & Higher maintenance (more frequent and complex maintenance)
**Performance**	**Adv**: Instant torque for quick acceleration & Quiet Operation **Dis**: Additional weight from batteries & Complex thermal management system	**Adv**: Mature technology with a proven track record & Extensive network of trained mechanics and availability of spare parts **Dis**: Higher noise pollution
**Initial Costs**	**Adv**: Lower long-term operational costs **Dis**: Higher initial purchase price due to battery costs & Battery replacement costs	**Adv**: Lower purchase price **Dis**: None
**Charging Infrastructure**	**Adv**: Potential for home charging **Dis**: Limited charging stations (especially fast-charging stations) & Longer charging times	**Adv**: Established and extensive fuelling network & Quick refueling. **Dis**: None
**Battery Limitations**	**Adv**: None **Dis**: Lower energy density (200–300 Wh/kg) compared to gasoline (12,000 Wh/kg), Performance degradation over time & Charging rate limitations due to electrochemical processes	**Adv**: Higher energy density **Dis**: No performance degradation due to fuel storage
**Design and Technical Challenges**	**Adv**: Potential for technological advancements in power electronics **Dis**: Additional weight from batteries & Advanced power electronics increase design complexity and cost	**Adv**: Simpler thermal management **Dis**: No significant weight from fuel storage
**Health and Safety**	**Adv**: Reduced noise pollution & Lower health risks from emissions **Dis**: Risks associated with battery fires & Health risks related to battery materials (e.g., lithium, cobalt)	**Adv**: None **Dis**: Significant health risks from emissions (respiratory issues, allergies, etc.)

To mitigate these environmental and health issues, there is a significant shift towards electric vehicles (EVs). EVs, which can be connected to the grid and recharged using clean, renewable energy sources, present a more sustainable alternative. The core of EVs is their battery storage systems, with lithium-ion (Li-ion) rechargeable batteries being the most widely used due to their high energy density and long range. Currently, the average cost of a Li-ion battery cell is around $100/kWh, but advancements in technology are expected to reduce this cost to $73/kWh in the coming decades, as reported by IHS Markit. Despite having a lower energy density (200–300 Wh/kg) compared to gasoline (12,000 Wh/kg), the high efficiency of electric propulsion systems ensures that EVs can still offer competitive driving ranges [1,2]. However, there are several challenges associated with the adoption of EVs. These include the high initial costs compared to internal combustion engine (ICE) vehicles, the limited availability of charging stations, and the need for substantial capital investment to develop a public charging infrastructure. Additionally, Li-ion batteries face issues such as performance degradation, limited energy density, and charging rate limitations due to electrochemical processes [3,4].

One major obstacle for EVs is the lack of charging stations, particularly fast-charging stations that can quickly recharge vehicle batteries, extending driving ranges and making EVs more practical for long trips. The development of a comprehensive EV charging network, similar to traditional petroleum stations, is essential, especially for long-distance travel. To address these issues, EVs can be integrated with smart grids to optimize their charging operations, enhance frequency regulation, and improve power usage efficiency, especially when combined with renewable energy sources (RES) [5]. Among RES, photovoltaic (PV) systems are particularly promising due to their productivity and advantageous investment costs [6]. A hybrid energy system that combines PV power, battery storage, and grid support can provide a robust and reliable charging infrastructure, compensating for the intermittent nature of solar energy [7,8]. In this investigation, we propose an innovative approach to significantly reduce the grid-tie capacity required for EV charging stations through the design of a common DC bus and an energy storage framework [9]. An optimized method is necessary to determine the ideal capacity for both the charging station and the energy storage system. We propose a cost-effective solution for AC/DC conversion that features a high power factor and low current distortion [10].

The integration of Global Maximum Power Point Tracking (GMPPT) with the converter design ensures optimal power extraction from the PV system, crucial for maintaining efficient energy conversion in charging stations. The single-stage topology simplifies the converter design, focusing on efficient DC–AC conversion, essential for feeding solar power into the grid or charging stations. The system provides power factor correction, harmonics filtering, and mitigation of power quality issues, which is crucial for maintaining stable and efficient charging station operations. With the control techniques, including various adaptive algorithms, can enhance the performance of charging stations by ensuring efficient power conversion and grid synchronization by the use of a single VSC. Existing control techniques might not perform well under adverse grid conditions, such as voltage fluctuations and distortions. Efficient operation requires a highly effective control technique for the DC–AC converter. More research is needed to develop control techniques that can handle all types of adverse grid conditions effectively. While various adaptive control algorithms have been proposed, their efficiency under abnormal grid conditions has not been comprehensively reported [11].

The use of advanced MPPT algorithms, such as the L-HC (Learning-based Hill Climbing) and HPO (Harmonic Perturb and Observe), ensures that the solar photovoltaic (PV) system operates at maximum power point (MPP), enhancing the overall efficiency of power generation. The converters employ adaptive control strategies like AM-MKF (Adaptive Modified Kalman Filter) and DFOGI (Delayed Signal Cancellation-based Frequency Locked Loop) for precise control of voltage source converters (VSCs). The use of converters with MPPT capability in charging stations allows for the efficient integration of solar PV systems, ensuring that maximum solar energy is harnessed and utilized for charging electric vehicles (EVs). By mitigating harmonics and ensuring a clean power supply, converters contribute to improved power quality at charging stations. This helps in protecting sensitive EV charging equipment and extending its lifespan. Converters can provide reactive power support and help in maintaining grid stability. This is particularly useful for charging stations connected to weak grids or those located in areas with high penetration of renewable energy sources. The ability to operate in different modes allows for seamless integration with energy storage systems. This can be used to store excess solar energy generated during the day for use during night time or peak demand periods, enhancing the overall efficiency and reliability of the charging station [12].

By employing efficient MPPT algorithms in the converters, charging stations can maximize the energy harvested from solar panels. This is particularly beneficial for off-grid and hybrid charging stations relying on solar energy. The fault ride-through capability of converters ensures that charging stations remain operational even during grid disturbances, providing a reliable service to users. The use of modular converter designs allows for scalable and flexible charging station configurations, accommodating different power levels and expanding as the demand for EV charging infrastructure grows. By leveraging these advantages, charging station designers can enhance the performance, reliability, and efficiency of their systems, ultimately providing a better service to electric vehicle users [13].

The voltage sensorless predictive control (VSPC) scheme eliminates the need for voltage sensors, reducing the overall cost of the controller. Integrated converters with reduced components for electric vehicles that utilize both solar and grid power sources are highlighted as an efficient design. The system is designed to be compatible with external battery management systems (BMS), ensuring additional safety and flexibility. The implementation of model predictive control (MPC) techniques can be complex, requiring detailed knowledge and precise tuning, which can be challenging under varying conditions. Some advanced converters support bidirectional energy flow, enabling not only charging but also discharging of EV batteries back to the grid, which is beneficial for grid stability and energy management. There is still a need for more robust control algorithms that can handle dynamic conditions such as partial shading more effectively [14].

Charging stations contribute to economic dispatch by balancing load demands and reducing operational costs. They facilitate the use of renewable energy sources by managing variable outputs and improving grid stability. Advanced control algorithms used in charging stations enhance the overall stability and reliability of the power grid. Charging stations play a crucial role in load management, peak shaving, and enhancing the flexibility of the power grid. They enable better integration of renewable energy sources, thus promoting a more sustainable energy ecosystem. There is a need for more robust cybersecurity frameworks to protect against potential attacks on the charging infrastructure [15].

The Parabolic Curve-fitting based Hill Climbing (PCHC) MPPT algorithm achieves an accuracy of 99.6 % in estimating the voltage corresponding to the maximum power point (MPP), which leads to increased system efficiency by reducing oscillations at MPP. The algorithm performs efficiently under varying environmental conditions and is capable of handling both sudden and slow changes in irradiance levels without losing the tracking direction. By optimizing the power output from the solar panels, the charging process for electric vehicles (EVs) becomes more efficient, leading to faster charging times and better utilization of the available solar energy. Implementing the PCHC algorithm might be more complex than traditional MPPT methods due to the need for continuous monitoring and calculation adjustments. While the PCHC algorithm improves efficiency and reduces oscillations, further research is needed to simplify the implementation and reduce computational requirements. Additional studies could explore the integration of the PCHC algorithm with other types of converters beyond the Boost converter to enhance its applicability in different scenarios [16].

Our survey of modern DC fast chargers highlights their superiority in electric fast charging (EFC) stations. We explore advanced power electronic converter frameworks suitable for EFC stations, focusing on both isolated and non-isolated DC/DC converter strategies. These strategies are implemented in bidirectional AC/DC front-end stages to meet the isolation requirements of traction batteries. We also discuss the advantages of replacing conventional line-frequency transformers with solid-state transformers (SSTs) in EFC systems. SST technology offers increased power density and performance, eliminating the need for low-frequency step-down transformers [17]. Table 2 outlines the merits, demerits, and benefits of charging stations, identifies research gaps, and evaluates the efficiency of the respective articles.

**Table 2:** It describes the advantages, disadvantages, and benefits of charging stations, highlights research gaps, and assesses the efficiency of the respective articles.

Ref	Advantages	Disadvantages	Benefits for Charging Stations	Research Gaps	Converter Used and Efficiency
[6]	**Revenue Generation:** The aggregator can maximize revenue by providing as much power capacity as possible when regulation prices are high. **Scalability:** The aggregator can manage small-scale power from individual vehicle batteries and provide large-scale power regulation services to the grid. **Cost Savings:** Vehicles connected to the grid can be paid for their available power capacity even when idle, similar to traditional generators. **Flexibility:** Vehicle batteries do not incur start-up or shut down costs, and their generation cost is trivial in frequency regulation due to the almost zero long-term mean of regulation requests.	**Complexity:** Designing an optimal aggregator requires careful consideration of various factors, including energy constraints, electricity prices, and regulation prices. **Battery Constraints:** The state-of-charge (SOC) limits the power capacity, and charging and discharging are restricted at the top and bottom of the SOC range to protect the battery. **Operational Challenges:** Regulation requests should not interrupt vehicles under charging, as it could distract the control loop of balancing supply and demand.	**Regulation Service:** Charging stations can provide frequency regulation services by managing the aggregated power of connected electric vehicles, thus contributing to grid stability. **Revenue Opportunities:** By integrating Vehicle-to-Grid (V2G) technology, charging stations can create additional revenue streams from the power capacity of idle vehicles. **Optimized Charging:** The dynamic programming algorithm ensures that the charging process is optimized for both cost and efficiency, taking into account fluctuating electricity prices and regulation demands.	**Real-World Validation:** Further research is needed to validate the proposed optimal control strategies in real-world scenarios and on a larger scale. **Consumer Behaviour:** Understanding the flexibility and behavior of vehicle owners regarding charging schedules and SOC requirements could improve the aggregator's performance. **Technology Integration:** Investigating how to seamlessly integrate V2G technology with existing grid infrastructure and market mechanisms remains an area for further study.	The proposed system ensures the optimality of charging control using dynamic programming, which balances the desired state-of-charge (SOC) level and profit from providing regulation power.
[8]	**Optimized Charging**: The method optimizes the charging schedules for each EV, considering both individual requirements and grid constraints, thus preventing grid congestion. **Grid Stability**: By avoiding power and voltage constraints, the system enhances the stability of the electricity grid. **Cost Savings**: Proper scheduling can reduce energy costs for EV owners by shifting charging to low-demand periods. **Environmental Benefits**: Efficient management of EV charging helps integrate renewable energy sources, reducing reliance on fossil fuels.	**Complex Implementation**: The system requires a sophisticated infrastructure for data collection, forecasting, and optimization. **Initial Costs**: Setting up the required communication and control systems can be expensive. **Data Dependency**: Accurate forecasting depends on comprehensive historical trip data, which may not always be available.	**Load Management**: Charging stations can manage loads better by distributing demand over time, reducing peak loads. **Revenue Opportunities**: Optimized charging can open new revenue streams through demand response programs and ancillary services like (V2G). **Enhanced Customer Satisfaction**: Ensuring EVs are charged as needed without causing grid issues improves customer trust and satisfaction.	**Forecasting Accuracy**: The document assumes perfect trip predictions, highlighting a gap in handling uncertainties in trip forecasting. **Scalability**: More research is needed to understand how the proposed methods scale with larger fleets and more complex grids. **Real-world Validation**: The need for extensive real-world testing to validate simulation results is emphasized.	It focuses on the general approach to managing EV charging through a centralized service provider (CSP), which implies the use of various types of converters to match grid and vehicle requirements.
[10]	**Grid Support Functions**: The PV/storage plant can participate in grid ancillary services such as frequency and voltage regulation. **Bidirectional Power Flow**: The system enables bidirectional flow of active and reactive power, which can stabilize the grid. **Enhanced Reliability**: Integration of batteries helps in decreasing the impact of PV source intermittency, providing a more stable power supply.	**Complex Control Systems**: The system requires sophisticated control mechanisms for managing multiple power sources and bidirectional power flow. **Initial Costs**: High initial investment for PV panels, batteries, and bi-directional converters can be a barrier. **Maintenance and Lifecycle**: Maintenance of batteries and power electronics can be complex and costly over time.	**Energy Storage**: The inclusion of batteries allows energy storage which can be used to charge electric vehicles during non-peak hours or when PV generation is not sufficient. **Grid Independence**: Charging stations can operate independently from the grid during outages by using stored energy in batteries. **Peak Shaving**: Reduces load during peak times by utilizing stored energy, thus reducing electricity costs.	**Scalability**: There is a need for research on the scalability of such systems to larger grids and varying geographic locations. **Efficiency Optimization**: Further studies are needed to optimize the efficiency of the converters and overall system under different operating conditions. **Economic Viability**: Research on cost reduction strategies and improving the economic viability of integrating PV, battery storage, and smart converters is essential	**Converters Used**: The system utilizes a boost converter for MPPT of the PV panels, a bi-directional DC-DC converter for battery management, and a three-phase inverter with delta modulation for interfacing with the grid. **Efficiency**: The efficiency of the DC-DC converters is implied through their ability to maintain a stable DC link voltage and perform efficient power conversion during both charging and discharging cycles.
[11]	**High Efficiency:** Single-stage topology is highlighted for its high efficiency due to minimal converter losses and low algorithm complexity. **Low Complexity:** The overall control complexity is reduced since only one voltage-source converter (VSC) is used. **Low Number of Switches:** Fewer switches are required in the system, simplifying the design and potentially reducing costs.	**Dependency on Control Technique**: Efficient operation requires a highly effective control technique for the DC–AC converter. **Performance under Abnormal Conditions**: Existing control techniques might not perform well under adverse grid conditions, such as voltage fluctuations and distortions.	**Power Quality Improvement**: The system provides power factor correction, harmonics filtering, and mitigation of power quality issues, which is crucial for maintaining stable and efficient charging station operations. **Unified Control System**: The use of a single VSC simplifies the control system, making it easier to manage and maintain. **Adaptability**: The proposed control techniques, including various adaptive algorithms, can enhance the performance of charging stations by ensuring efficient power conversion and grid synchronization.	**Performance under Adverse Conditions**: More research is needed to develop control techniques that can handle all types of adverse grid conditions effectively. Current techniques may not be suitable for all scenarios. **Algorithm Efficiency**: While various adaptive control algorithms have been proposed, their efficiency under abnormal grid conditions has not been comprehensively reported.	**Converter Type**: The document discusses the use of a three-phase VSC for DC–AC conversion. **Efficiency**: The proposed method using a single-stage topology is highlighted for its high efficiency.
[12]	**Learning-based Hill Climbing (L-HC) MPPT Algorithm:** Mitigates oscillations in steady-state conditions. Provides a faster response during dynamic changes. Ensures operation at maximum power point (MPP) under varying solar irradiation conditions. **Adaptive Maximize-M Kalman Filter (AM-MKF):** Capable of handling unity power factor (UPF) operation and DSTATCOM operation at night. Provides accurate harmonic supportive control, reducing nonlinear current exchange with the grid. Maintains power quality and sinusoidal nature of grid currents. **Grid-Tied Solar PV System**: Provides power to the grid during the day and supports reactive power at night. Handles unbalanced loads and variable solar insolation effectively.	**Complexity:** The control schemes (L-HC and AM-MKF) are complex and require precise implementation and tuning. **Dependency on Accurate Sensing:** Requires accurate current and voltage sensing for optimal performance, which may increase system cost and complexity.	**Improved Power Quality:** The integration of advanced control techniques ensures high-quality power delivery to the grid, beneficial for charging stations requiring stable and reliable power. **Efficient Energy Utilization:** Maximum Power Point Tracking (MPPT) ensures that the maximum possible energy is harvested from the solar PV, optimizing the charging process for electric vehicles (EVs). **Versatility in Operations:** The ability to operate in both UPF and DSTATCOM modes allows the system to provide support during both daytime and night-time, enhancing the overall reliability of charging stations.	**Dynamic Performance under Variable Conditions:** Further research is needed to improve the performance of control algorithms under highly dynamic conditions and extreme weather variations. **Simplification of Control Schemes:** Simplifying the complex control algorithms without compromising performance could make the system more accessible and cost-effective. **Long-term Reliability and Maintenance:** Studies on the long-term reliability and maintenance requirements of these advanced control systems in real-world conditions are limited and warrant further exploration.	**Converters Used:** **Boost Converter**: Used for MPPT, controlled by the L-HC algorithm. **Voltage Source Converter (VSC):** Used for AC to DC conversion, controlled by the AM-MKF algorithm. **Efficiency:** The system's performance, including the converters, ensures low Total Harmonic Distortion (THD) and efficient power conversion.
[13]	**Efficiency in Power Conversion**: The system uses a DC-DC converter for MPPT, ensuring the solar PV modules operate at maximum efficiency. **Reduced Oscillations**: The L-HC algorithm mitigates issues like oscillations in steady-state conditions and slow response during dynamic changes. **Adaptive Control**: The AM-MKF ensures optimal performance under various conditions, including unbalanced loads and variable solar insolation.	**Complexity in Implementation**: The use of advanced algorithms like L-HC and AM-MKF can increase the complexity of the system design and implementation. **Cost**: Implementing such sophisticated control mechanisms may lead to higher costs compared to simpler systems.	**Improved Power Quality**: The system provides reactive power support during night-time, maintaining power quality and stability. **Enhanced Efficiency**: By using advanced MPPT algorithms and adaptive control techniques, the charging station can achieve higher efficiency and reliability. **Flexibility**: The system can adapt to various adverse grid conditions, making it robust and reliable for continuous operation.	**Real-World Testing**: While the document shows prototype testing, more extensive real-world testing under various environmental and load conditions could further validate the system's performance. **Cost-Effectiveness**: Research into reducing the cost of implementing such advanced control systems without compromising performance could be beneficial.	**Converters Used**: The system utilizes a boost converter for MPPT and a Voltage Source Converter (VSC) for AC-DC power conversion. **Efficiency**: The system indicates a Total Harmonic Distortion (THD) of 2.4 % for grid current during power feeding, which signifies good efficiency in power conversion.
[14]	**Cost Reduction**: The proposed voltage sensorless predictive control (VSPC) scheme eliminates the need for voltage sensors, reducing the overall cost of the controller. **Enhanced Response**: The VSPC scheme improves the dynamic response of the system, particularly during changes in irradiation. **Reduced Component Count**: Integrated converters with reduced components for electric vehicles that utilize both solar and grid power sources are highlighted as an efficient design. **Compatibility with External BMS**: The system is designed to be compatible with external battery management systems (BMS), ensuring additional safety and flexibility	**Complexity**: The implementation of model predictive control (MPC) techniques can be complex, requiring detailed knowledge and precise tuning. **Dependence on Accurate Modeling**: The effectiveness of predictive control schemes depends heavily on accurate system modeling, which can be challenging under varying conditions.	**Improved Efficiency**: Converters are crucial in enhancing the efficiency of energy transfer from photovoltaic (PV) arrays to electric vehicle (EV) batteries, ensuring MPPT under various conditions. **Bidirectional Energy Flow**: Some advanced converters support bidirectional energy flow, enabling not only charging but also discharging of EV batteries back to the grid, which is beneficial for grid stability and energy management. **Reduced Power Losses**: By optimizing the power conversion process, these converters minimize losses, making the overall system more energy-efficient and cost-effective	**Dynamic Condition Handling**: While significant progress has been made, there is still a need for more robust control algorithms that can handle dynamic conditions such as partial shading more effectively. **Integration with Emerging Technologies**: Further research is needed to integrate these converter technologies with emerging smart grid and IoT frameworks to enhance their functionality and interoperability. **Scalability and Implementation**: Practical implementation and scalability of these advanced converters in real-world charging stations remain an area requiring more research and development.	**Converters Used**: A MPPT dc–dc boost converter is used. **Efficiency**: Is more than 99 % proves the suitability for industrial application.
[15]	**Economic Dispatch (ED) Optimization**: Charging stations contribute to economic dispatch by balancing load demands and reducing operational costs. **Integration with Renewable Energy**: They facilitate the use of renewable energy sources by managing variable outputs and improving grid stability. **Reduction in Emissions**: Electric vehicle (EV) charging stations help lower greenhouse gas emissions compared to traditional fossil fuel-based transportation. **Support for Grid Stability**: Advanced control algorithms used in charging stations enhance the overall stability and reliability of the power grid.	**Cybersecurity Risks**: The integration of information and communication technologies introduces vulnerabilities to cyber-attacks, such as false data injection attacks (FDIAs). **High Initial Costs**: The installation and setup of charging stations require significant upfront investment. **Dependency on Technology**: Over-reliance on advanced technologies can lead to issues if there are technical failures or insufficient maintenance.	**Cost Savings**: By optimizing energy use, charging stations can lower the costs associated with electricity consumption. **Environmental Impact**: Reduced emissions from EVs help in meeting environmental regulations and promoting sustainability. **Grid Management**: Charging stations play a crucial role in load management, peak shaving, and enhancing the flexibility of the power grid. **Renewable Integration**: They enable better integration of renewable energy sources, thus promoting a more sustainable energy ecosystem.	**Cybersecurity Measures**: There is a need for more robust cybersecurity frameworks to protect against potential attacks on the charging infrastructure. **Cost Reduction Strategies**: Research is required to find ways to lower the initial costs and make charging stations more economically viable. **Technological Innovations**: Further development in converter technologies and grid integration methods to enhance efficiency and reliability.	It emphasizes the importance of high-efficiency converters that can handle variable power demands and integrate with smart grid technologies. Typically, DC-DC converters are widely used in EV charging stations due to their high efficiency and capability to manage power flow effectively.
[16]	**Reduced Oscillations**: The PCHC algorithm reduces steady-state oscillations compared to conventional MPPT techniques, improving both steady-state and dynamic behavior of the system. **Cost-Effective**: The reduced sensor approach using only one current sensor significantly lowers the controller cost, making it economical for rooftop solar PV systems. **Adaptability**: The algorithm performs efficiently under varying environmental conditions and is capable of handling both sudden and slow changes in irradiance levels without losing the tracking direction.	**Complexity**: Implementing the PCHC algorithm might be more complex than traditional MPPT methods due to the need for continuous monitoring and calculation adjustments. **Dependence on Accurate Measurements**: The effectiveness of the algorithm heavily relies on the accuracy of current and voltage measurements, which might require high-quality sensors.	**Improved Charging Efficiency**: By optimizing the power output from the solar panels, the charging process for electric vehicles (EVs) becomes more efficient, leading to faster charging times and better utilization of the available solar energy. **Cost Savings**: Lower sensor and controller costs make it more feasible to deploy efficient charging stations in residential and commercial settings. **Sustainability**: Enhancing the efficiency of solar power conversion supports the deployment of more sustainable and environmentally friendly EV charging infrastructure.	**Enhanced Algorithms**: While the PCHC algorithm improves efficiency and reduces oscillations, further research is needed to simplify the implementation and reduce computational requirements. **Integration with Various Converter Types**: Additional studies could explore the integration of the PCHC algorithm with other types of converters beyond the Boost converter to enhance its applicability in different scenarios. **Long-Term Performance**: Research on the long-term performance and reliability of the PCHC algorithm in diverse environmental conditions would be beneficial.	**Converter Used**: The system uses a Boost converter, as mentioned in the validation of the proposed method through simulations and hardware prototypes. **Efficiency**: The proposed PCHC MPPT algorithm achieves an efficiency of around 99.6 % in tracking the MPP, significantly improving the overall efficiency of the solar PV system.

This article presents a comprehensive survey of various multiport converters with RES integration for EFC in EVs. It is evident that the development of ultra-fast charging stations is a critical area for future research and development. Overcoming these barriers is essential for the successful growth and maturation of the EV industry.

A. **Charging Cable Technology**: Develop advanced charging cables that can handle high power levels required for ultra-fast charging while ensuring safety and efficiency. Research should focus on materials, insulation, and connector design to minimize heat generation and energy loss.
B. **Protection Devices**: Design and implement robust protection devices to safeguard both the EVs and charging infrastructure. This includes overcurrent protection, voltage regulation, and surge protection mechanisms to ensure safe and reliable charging.
C. **Efficient Power Converter Design**: Research and develop efficient power converter designs, possibly using wide-bandgap semiconductor devices such as silicon carbide (SiC) and gallium nitride (GaN). These technologies can handle high power levels with lower losses, making ultra-fast charging more energy-efficient.
D. **Solid-State Transformers**: Explore the use of solid-state transformers in charging stations to improve the overall efficiency and reliability of power conversion. Solid-state transformers can offer advantages in terms of size, weight, and controllability compared to traditional transformers.
E. **Integration of Photovoltaics (PV):** Investigate the integration of solar panels (PV) into charging stations to harness renewable energy sources. This can reduce the environmental impact of charging and make EV charging stations more sustainable.
F. **Energy Storage Integration**: Explore the integration of energy storage systems (e.g., batteries or supercapacitors) into charging stations. This can help mitigate peak power demands, improve grid stability, and provide backup power during outages.
G. **Partial Power Converters**: Continue research into partial power converters that process only a fraction of the full available power. This approach can help optimize charging efficiency and adapt to varying charging requirements, making charging infrastructure more flexible and cost-effective.
H. **Standardization and Interoperability**: Collaborate with industry stakeholders to establish common standards for ultra-fast charging technology and ensure interoperability between different EV models and charging station networks.
I. **User Experience**: Consider the user experience in the design of ultra-fast charging stations. This includes user-friendly interfaces, payment systems, and accessibility features to encourage the adoption of EVs.
J. **Grid Integration**: Study the impact of ultra-fast charging stations on the electrical grid and develop smart grid solutions to manage power distribution and prevent grid congestion.
K. **Environmental Impact Assessment**: Conduct comprehensive environmental assessments to understand the ecological footprint of ultra-fast charging infrastructure and identify ways to minimize its impact on the environment.

By focusing on these research areas, we can work toward overcoming the barriers to the successful growth of the EV industry and provide users with convenient and efficient ultra-fast charging solutions.

The paper is organized into several sections, beginning with Section II, which discusses the framework of EV fast charging stations. In Section III, the paper delves into the integration of renewable energy sources (RES) for electric vehicle charging stations, exploring their benefits and current industry trends. Section IV focuses on power electronic converters, specifically addressing their integration with RES in the context of DC charging stations. Section V introduces an SST-based EFC architecture for medium voltage (MV) applications. Finally, Section VI offers insights and forecasts for the future development of EFC, particularly in the context of multiport converters for electric vehicles. The paper concludes in Section VII.

## 2 EV fast charging station framework

According to the Society of Automotive Engineers (SAE) standards, from SAE J1772 standards the conductive charging can be balanced in the USA for both EVs/Plug in Hybrid Electric Vehicles (PHEV) [18]. Owing to the lack of curtailment charging infrastructure, customers are urged to charge their EVs through residential networks. According to SAEJ1772 standards, these chargers are specified as level-1 having 120 V and level-2 having 240 V [19]. Onboard chargers for AC level-1 as 120V and AC level-2 as 240V ac inputs, will deliver a peak power of 1.9 kW and 19.2 kW accordingly. The dc fast chargers can be implemented in an onboard chargers for limited power ratings typically 50 kW. At present, the power levels are increased to 475 kW. These chargers can be delivered dc power through isolated power electronic converters for an EV battery charge and it provides high-speed charging.

In EVTECH manufacturing, espresso&charge can be designed for up to 150 kW DC and 65 kW AC. It resists up to a voltage of 1 KV for fast charging standards. The Output power is controlled sensibly while parallel charging, therefore connected EVs are optimally catered for an instant time, it is known as “Dynamic DC-power splitting”. Features for an espresso&charge are less impact on the grid, remote access and 4 vehicles can be charge parallel at the same time. In ABB manufacturing, HP Terra chargers are fast charging with a power rating of 350 kW and it withstand an output voltage of 920V dc and output current 500A. It is more flexible with a cost-efficient to build comfortable charging points that will increase the demand in the EV market. Energy management can be achieved for an Open charge point protocol smart charging. The Phihong DO series has a multi-standard charging connectors like CHAdeMO, CCS, and GB/T protocols. It has 360 kW output power, 500V dc output voltage, and an output current of 150A and it can be charge 4 DC charging points simultaneously. It supports pantograph charging and load balancing. Main applications include EV bus stations and highway gas stations.

Near attractive facilities such as cafes, shopping, and WiFi hot surfaces, Tesla Supercharger stations are conveniently located. To travel easily on the road, each station includes several superchargers. An additional feature can be applied under the Supercharger framework to the Tesla vehicles are described below. Owners are billing per kWh (kilowatt-hour) where possible, which is the fairest and easiest process. It can charge per minute in other places. Two ranges accommodate for shifts in charging rates, called 'tier 1' and 'tier 2' while paying per minute. Tier 1 applies when vehicles are loaded at below 60 kW and tier 2 applies when vehicles are loaded over 60 kW. Tier 1 is half of the tier 2 cost. Tier 1 also occurs if the vehicle shares the capacity of the supercharger with another drive.

The Tritium Veefil PK350 is a 350 kW Level 3 DC fast charging station with dual ports- CHAdeMO and SAE Combo connector (CCS). A scalable and flexible DC fast charge solution, the Tritium Veefil PK350 can serve many EV drivers at the same time. The system unique advantages include small footprint, scalability, flexibility, and high efficiency. Environmentally safe (water-based liquid cooling). It Offer both CHAdeMo and CCS Proven liquid cooling inside the user unit ensures the longevity of the power electronics and leads to high level efficiency.

**Fig:1:**
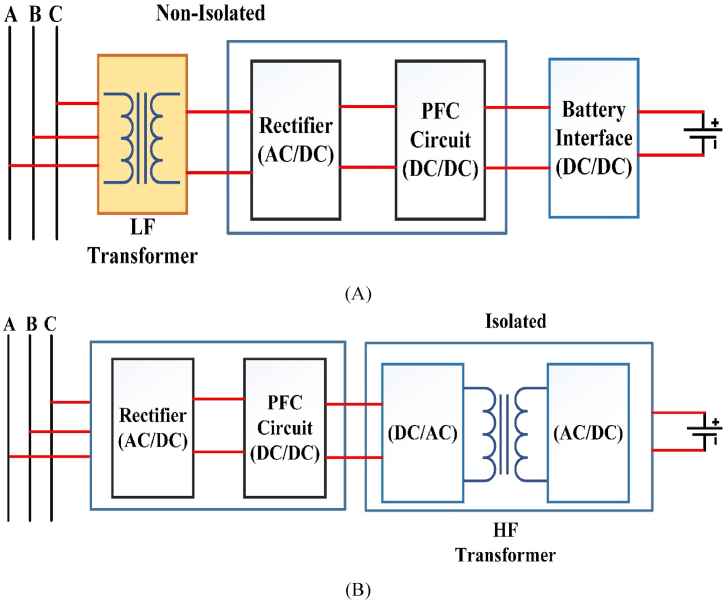
Single - line charger system with (A) Non-Isolated converter (B) An Isolated converter.

Table 3 briefly explains the dc fast charger state-of-the-art for EV batteries. To attain 920v dc at output side power electronic converters plays a major role with an input of 480V ac. In case of conventional converters required two-stage of operation that is: the first conversion is ac to dc and the second one is dc to dc conversion, which requires more switches. while using more switches, switching losses and efficiency of the converter is decreases. Multiport converters are suitable for interconnecting with RES. A single-line diagram of non-isolated and isolated converters is shown in Fig. 1(A) and Fig.(B) respectively.

**Table:3:** Current manufacturing companies DC fast Chargers state-of-the-art.

Manufacturer	Model Type	Input Voltage	Output Voltage	Output Current	Power(kW)	Efficiency	Cooling System	Supported Protocols	Temperature Range	Time to Charge
Tesla	Supercharger	480V AC	50–410V DC	330A	140	91 %	Liquid	V3 Superchargers	−30 °C–50 °C	30 min
EVTEC	Espresso & Charge	400V AC	500V DC	300A	150	93 %	Less Ventilation	CHAdeMO JEVS	−20 °C–50 °C	24 min
PHIHONG	DO Series	415V AC	500V DC	150A	360	>94 %	Fan	CHAdeMO V1.2	−30 °C–50 °C	15 min
ABB	Terra HP	480V AC	920V DC	500A	350	95 %	Liquid	CHAdeMO CCS-1	−35 °C–55 °C	10 min
Tritium	Veefil PK	480V AC	920V DC	500A	475	>98.5 %	Liquid	CHAdeMO	−35 °C–50 °C	10 min

For the co-development of the next-generation ultra-fast EV charging standard, CHAdeMO signs an MoU with China Electricity Council (CEC). In order to do not penalize the existing EV users, the latest ultra-fast charging standard would ensure backward compatibility with both current CHAdeMO and GB/T standards. The next-generation ultra-fast charging technology that is stable and flexible can lead to this joint growth. Since the latest Ultra-Fast Charging Standard aims to ensure interoperability with current fast charging standards for CHAdeMO and GB/T, it is also expected to be implemented not only in Japan and China, but also in several other EV markets around the world, and leads to the next implementation of the EV charging network. Different types of fast charging modules with specific advantages are mentioned in Table 4.

**Table 4:** DC fast charging standard models and specifications.

Standard	GB/T	Telsa	CCS-1	CCS-2	CHAdeMO 2.0	CHAdeMO 3.0
Power	120 kW	135 kW	150 kW	175 kW	400 kW	900 kW
Maximum Voltage	1 kV	410V	600V	1 kV	1 kV	1.5 kV
Maximum current	250A	330A	200A	200A	400A	600A
Country	China	All markets except the EU	North America	EU and rest of markets	Japan	China
Protocol	CAN Bus	Hybrid CAN bus and J1772	Programmable Logic Controllers (PLC)	PLC	Control Area Network (CAN) Bus	CAN Bus
Coupler Inlet						

Wireless charging (WC) is an alternative approach to the EFC stations, which fully avoid the charging cable. Inherent galvanic isolation and convenience include other benefits of WC. However, typically these systems having less performance and has lower power density over conductive charging systems [[20], [21], [22]]. The study and discussion of wireless charging technology are outside the scope of this report.

Table 5 explains the different types of EV models with driving range, battery capacity, and energy cost per kilometre [[23], [24], [25], [26], [27], [28], [29], [30], [31], [32], [33]]. For Specifications commercially available chargers concerning engine rating, type of engine, battery capacity, and electrical range. It can be clear that further research efforts have been fetched out to make EVs has less cost-effective while providing more than 200 miles of electrical coverage.

**Table 5:** Comparison between old and latest EV Models concerning Driving range, Energy cost/Km and mainly it describes the Battery capacity.

EV Model	Driving Range (km)	Battery (kWh)	Energy cost/Km ($)
Mitsubishi MiEV	85	16	0.038
Honda Fit	112	20	0.036
Ford Focus	110	23	0.04
Mercedes B	136	28	0.04
Nissan Leaf	160	30	0.038
BMW i3	345	42	0.033
Tesla S 60	275	60	0.044
Tesla 3	496	75	0.030
Tesla S 85	360	90	0.048

**Fig. 2:**
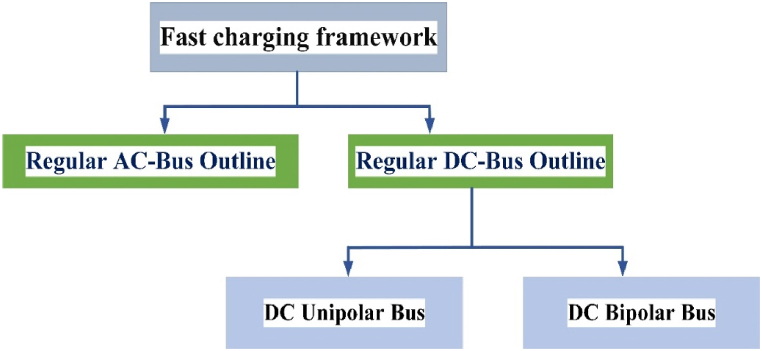
Classification of Fast charging station framework.

The EV batteries can be recharged with the grid by AC or DC chargers. To allow these high charging rates, different connectors have been used globally. Currently used connectors worldwide with their individual strength rating [34,35]. These methods have been described in the below sub-chapters. The EV fast chargers can be emulate the gasoline station infrastructure with the feature of delivering multiple EVs, the architecture and configuration are crucial. The two potential configurations of EV chargers are recognized in Ref. [36] and their classifications are shown in Fig. 2.

Table 6 demonstrates the charging levels along with their grades like sluggish, quick and extreme fast charging requirements. To allow high charging speeds, different connectors are used globally.

**Table 6:** Charging power levels of AC and DC types.

Charging Modes	Voltage ratings	Power ratings (kW)	Charging Time
Mode 1 (Slow)	120Vac	3.7	10–15 h
Mode 2(Slow)	220Vac	3.7–22	3.5–7 h
Mode 3 (Fast)	3-Ø 480Vac	22–43.5	10–30 min
	200-600Vdc	*<200*	
		<150	

## 3 Integration of RES for an EFC stations: benefits and current trends

The Charging costs are directly proportional to its speed. With this economic reasons, the fast charging rates exceeds the conventional charging rates are quite relevant. In modern years there is a large disposition with fast chargers over the world. Since EVs will require more power from the grid-related to the other loads, due to large power the harmonics are occurred at grid side. One of the research project in European Union passenger vehicle fleet that 80 percent of EVs are consuming 95.5 % power from the complete grid side [37]. There is a need to update traditional delivery systems and transformers to meet the requirement of fast charging. Moreover, the integration of multiple EV chargers has a negative impact on grid stability. A sustainable low-cost and efficient ecosystem is created by the use of smart meters to monitor charging. To witness that the grid is very useful with significance of smart charging methods. The installation costs of dc fast chargers vary from location to location, a large portion of area comes from the improvement of electrical services [38]. It makes more economic to build EFC charging stations with several chargers than to build single-port chargers because few of the overhead site construction is distributed over multiport charging. With multiport chargers sharing a similar upstream infrastructure, allowing installations in urban areas, the size of EFC stations per channel can be reduced dramatically. Unless the EV charge is left unregulated, due to EV charging, there may be a normal peak load increment, substantial peak load shifting, feeder overloading, escalating transformer aging, and also increases the power losses [[39], [40], [41]].

EFC stations of various RES and battery energy storage (BES) systems are introduced in Refs. [42,43], is one possible way of solution to minimize the need for electricity and degrading the effects of vehicle charging in the grid network. The EFC station can also be the interconnecting platform with RES such as photovoltaic, biomass, and wind [44]. In addition to adding generation to the station and reducing tariffs, incorporation RES and exploiting V2G topology often allows the power transmission among charging station, grid to be profiled, thus offers grid services like load shifting and frequency regulation [45,46], voltage control reactive power support [47,48], and reinforcing renewable generation [[49], [50], [51]]. It is also possible to change the charging capacity of the vehicle according to the convenience of RES [52,53]. Wind, solar, or some other energy source could be involved. Besides charging time and the bidirectional power flow can be controlled by the smart charging methods. It's not only improves the productivity, but it also assist to diminish the peak demand for the distribution grid [54]. In addition to this, solar and wind-generated power will make electric vehicles even more sustainable. The vehicle-to-everything (V2x) technology enables the storage battery power flow from vehicles to residential and other loads [55]. The furthermost advantage of the V2x topology is that it allows electricity to be stored, especially from RES. By using these storage devices, the peak power demands can be met. It is also worth noting that because of the technical constraint, V2G is a major challenge to use AC chargers as on-board bi-directional communication stages are required.

To modify the charging power by focusing on the grid ability and regional power demand from various loads, the local load balancing solution is simple as moving the charging time slot [56]. In case, if the smart charger senses that the power from other load applications has decreased, it can boost the charging power in a controlled manner. Conversely, the capacity of the charger is limited to offset the load if the local load demand rises. Another approach is to balance the charging methods of several facts towards a priority consideration. With priority or with modified power, smart chargers can be triggered sequentially [57,58]. By using BES with the required control technique, the intermittency of the RES can also be enhanced. Besides, V2G technology will allow individuals to use their vehicles as per enormous energy storage to assist the disproportion grid during peak loads. All-electric vehicles have been described earlier, the energy storage functioning is enormous from the grid perspective. If there is excess power from RES with the development of modern optimization techniques, electric cars could be charged, and this energy can send back to the grid using V2G technologies. It helps to change the peak power of both consumption and generation, thereby filling the gap in supply demands. In the future, EVs will serve as backup power generators. EVs can be used to energize the local loads in the event of a grid failure for less period, thereby supplying emergency power during an outage. A greater number of apparatus especially power electronics (PE) parts decreasing the overall system's reliability. Large numbers of PE control switches often necessitate large quantities. The number of drive circuits that add to the drive in turn device complexity and reduces reliability. The difficulty of control methods also adds to the complexity of control policies, Reduction of overall reliability.

### 3.1 AC fast charging station

**Fig. 3:**
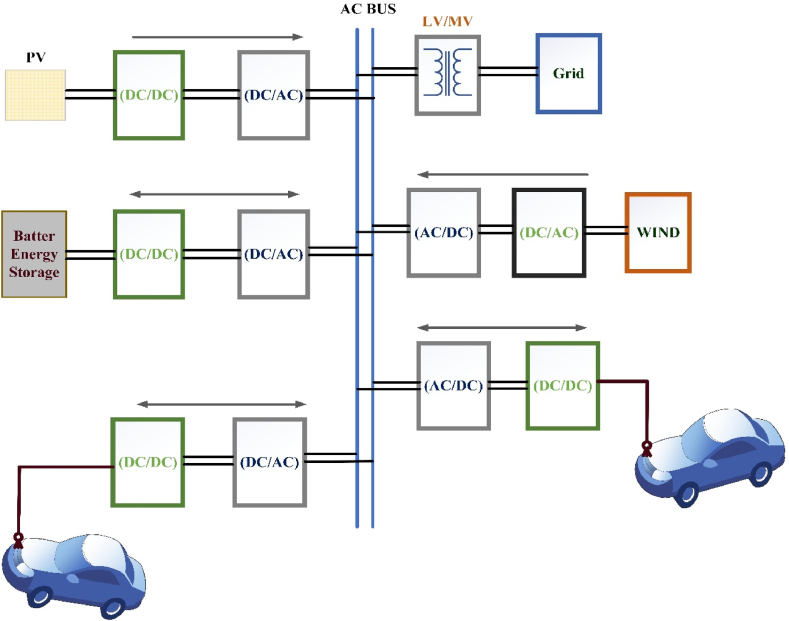
AC interface charging station.

For an ac-connected appliance, a step-down transformer is interfacing among the distribution network and a 3-Ø ac bus system to operate at a 250–480V line-to-line voltage. This ac bus is providing each charger at the stations and each charger appearance an independent ac-dc stage. This method elaborates the numerous stages in conversion among the distributed network and the EV dc terminal or RES. In an ac-connected system, having more conversion stages it increases the complexity and expense of the system and decreasing the performance of the system. The benefits of using ac bus include the availability, sophistication of rectifier and inverter technologies, the feasibilities with an ac switchgear for protects the devices, and well-established specifications for an ac power delivery system. Besides, EV charging point standards such as in Refs. [[59], [60], [61]] are being established. As seen in Fig. 3, ac-connected networks are the majority of modern EFC stations. In Mountain View, California, Tesla superchargers are used and in Europa, Victoria, ABB are using dc fast-chargers [62].

Despite having a universal AC socket, there is no limitation on charging the EV. It is mainly built with the EV manufacturer of the company, power levels, frequency levels, and country. It is designed with various shapes, volumes, and pin-out. At present 4 types of AC charging connectors are employed in the worldwide. Type-1 connector standard SAEJ1772 is a single-phase charging and it contains 5 pins: 2 pins for AC lines, 2 pins for signal line, and one pin are for shielding ground path. The peak voltage is rated up to 120V or 240V and current is withstand up to 80A. Type-2 connector standard International Electrotechnical Commission(IEC) IEC 62196-2 is suited for both AC and DC charging, it is suitable of 3-phase AC system also, the ratings of voltage and current of systems are 1-phase 230V,80A, and 3-phase 400 V, 63A respectively. Type-3 connector standard GB/T 20234 is capable in both AC and DC charging used for a 3-phase system with a voltage of 380V and current of 35A. Type-4 is a Tesla US connector it is handling both AC and DC charging and is specifically used in the US states. The peak ratings of voltage and power are 240 Vac and 17.2 kW charging.

### 3.2 DC fast charging station

**Fig. 4:**
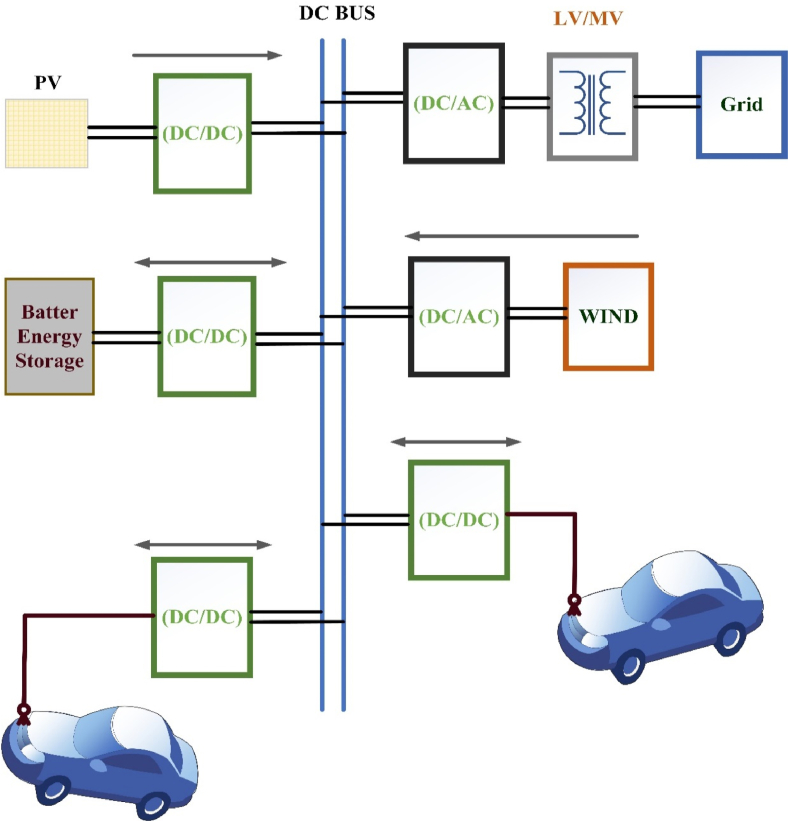
DC interface charging station.

The dc bus design requires a central front-end ac-dc converter for the dc-connected network which offers a high energy-efficient path to interconnect both RES and dc storage. Voltage levels at this point, the architecture of the dc bus EFC stations should comply with the same requirements as that of the AC bus EFC station. Among the dc bus and a dc-dc converter, each charger is interconnected, eliminating the individual ac/dc converters. The system performance is enhanced compared to the ac-connected systems with a decreased number of conversion levels. One possible benefit for the "dc distribution" strategy is that the central front end provides a mono interconnection to the utility. The complexity of control schemes can be easily simplified due to the lack of reactive power in dc systems [63]. The partial power converters has been handled an amount of electricity supplied to the vehicle, degrade the converter ratings, minimize the cost of the system and enhance the conversion efficiency. In Refs. [64,65] suggest the power dc-dc converters communicate with the standard dc bus of an EFC station based on specific needs as seen in Fig. 4.

These are intended to supersede chargers of Level 1 and Level 2. Depending on the manufacturer, they are rated from 50 kW to 500 kW. The power conversion and the control stage are more bulky and costly with higher power volume. This is one of the key reasons for the off-board implementation of DC fast chargers. The other reason for selection dc chargers is for protection purpose. Security of the passengers becomes a key problem with large power converters and enlarged the size of power handling apparatuses. In Table 7 briefly explained the ac and dc tied EV charging station system with merits and demerits for respected parameters.

**Table 7:** Assessment among AC and DC tied EV system.

Parameters	AC Interface system	DC Interface system
Conversion modes	High	Less
Complexity	High	Less
Availability	High	Less
Cost	High	Less
Technical Maturity	High	Less
Protective devices	Less	High
Grid Operation at abnormality	High	Less
Efficiency	Less	High

## 4 Power electronic converters with integration of RES for DC charging stations

**Fig:5:**
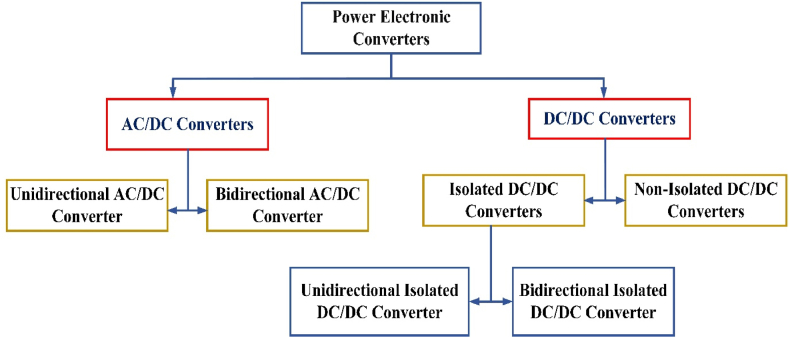
Classification of Power electronic converters for EV Charging stations.

In the literature, several power electronic converter topologies for charging stations are suggested as shown in Fig. 5, that can provide some of the above features and can be categorized as follows.

### 4.1 Bidirectional AC/DC converters

**Fig. 6:**
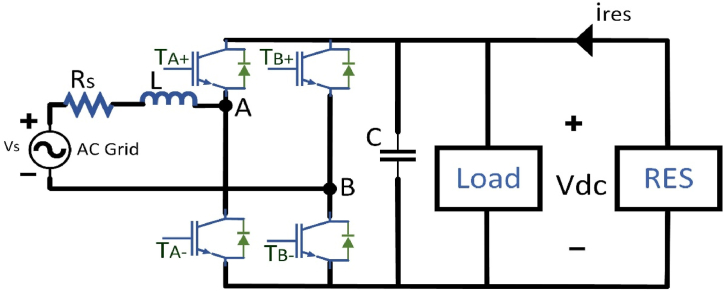
Bidirectional AC/DC converter in RES.

The bidirectional ac/dc converter plays an important role in the renewable energy system. It is used as the interface between Distributed energy resources and the AC grid system as shown in Fig. 6. It can be deliver power in bidirectional, enhance the ac current and good dc voltage regulation by using a PWM techniques. Harmonics can be eliminates by effective current shaping in an ac grid system, and it provides a high quality dc load with good dc voltage regulation. To accomplish both charging and discharging of the ac side inductor current, a simplified PWM has adjusts one active switch status during the switching time. As a result, the simplified PWM technique eliminates switching losses and it provides high conversion efficiency. Table 8, Table 9 show the switching states of the simplified PWM in rectifier mode and inverter mode operation, correspondingly [66].

**Table 8:** Rectifier Mode switching operations.

Source voltage	TA_+_	TA-	TB_+_	TB-	Inductor Status
V_S_ > 0	OFF	OFF	ON	OFF	V_L_ > 0
	OFF	ON	OFF	OFF	
	OFF	OFF	OFF	OFF	V_L_ < 0
V_S_ < 0	ON	OFF	OFF	OFF	V_L_ < 0
	OFF	OFF	OFF	ON	
	OFF	OFF	OFF	OFF	V_L_ > 0

**Table 9:** Inverter Mode switching operations.

Source voltage	TA_+_	TA-	TB_+_	TB-	Inductor Status
V_S_ > 0	ON	OFF	OFF	OFF	V_L_ > 0
	OFF	OFF	OFF	ON	
	ON	OFF	OFF	ON	V_L_ < 0
V_S_ < 0	OFF	ON	OFF	OFF	V_L_ < 0
	OFF	OFF	ON	OFF	
	OFF	ON	ON	OFF	V_L_ > 0

**Fig. 7:**
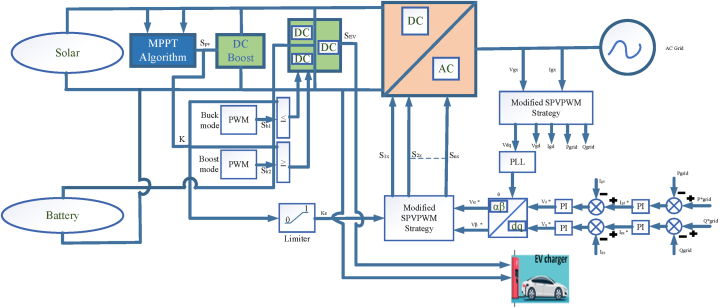
Control scheme for simplified PWM technique.

A single-phase bidirectional ac/dc converter cannot operate adequately with the conventional dual-loop control scheme added to the simplified PWM. To accomplish power factor correction in ac side and voltage management in dc side, a PI controller is used in the voltage controller and current controller in general. To yield fast output voltage and better current shaping response control capability can be enhanced by the feedforward control signal. Thus, the developed simplified PWM control scheme is shown in Fig. 7. It is notable on feedforward control scheme is ideal for both the simplified PWM strategy and the traditional Bidirectional PWM and Unidirectional PWM strategies.

### 4.2 Bidirectional DC/DC converters

**Fig. 8:**
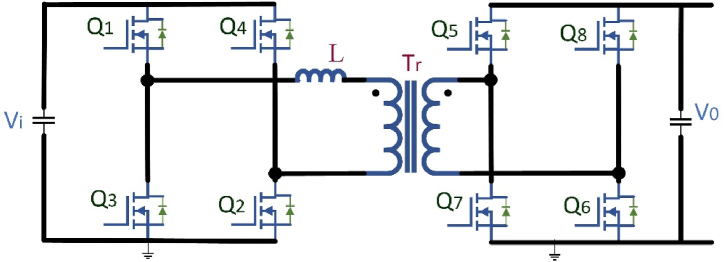
Bidirectional DAB

High power density, high energy exhaustion, broad gain spectrum, galvanic isolation, and bidirectional power flow can be achieved by the Dual Active Bridge (DAB) and capacitor-inductor-inductor-capacitor (CLLC) dc-dc converter topologies hence potential dc-dc converters has bidirectional power flow uses. A DAB converter is shown in Fig. 8 for bidirectional power flow. Owing to its large power density, maximum reliability, buck and boost converter performance, low system tension, minor filter components, and reduced sensitivity to system variance can used for EV charging stations. More recently, the DAB converter has began to attract interest, allowing converter performance and power density enhancements owing to the versatility of the latest SiC and GaN-based power electronic circuits and development in Nanocrystalline and shapeless soft magnetic substances [67]. The power flow is regulated in the DAB converter by changing the phase shift among the primary and secondary side voltage, with the power-transfer portion acting as the transformer leakage inductance. Because of its solid approach and Zero Voltage Switching (ZVS) working [68,69], the DAB converter is being widely used during isolated bidirectional dc-dc conversion systems. To work with a broad array of voltage gain and energy, the converter is required for EV battery charging owing to the EV charging configuration in which the reactive power can be increase drastically and does not hold a longer state for ZVS [70].

The Dual-Phase Shift (DPS) modulation is designed in Ref. [71] to diminish the current burden of switching units, whereas an additional freedom of degree to the primary and secondary service cycles are added. The DPS is arrogated in Ref. [72] to accomplish ZVS under maximum load span. The definition of DPS is supplementary expanded to Triple Phase Shift (TPS) in Refs. [73,74] to allow for more notches of choice and numerous design goals, such as a wider state of ZVS, lower current tension, and enhanced performance. Recent research in Ref. [75] uses TPS to increase the performance of light loads while switching to DPS to minimize the circulating current under moderate and higher load situations.

**Fig. 9:**
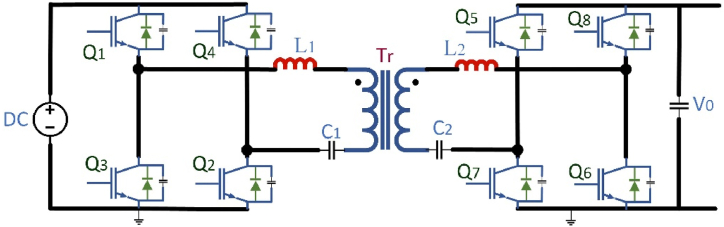
Full bridge bi-directional CLLC resonant converter.

In DC distribution applications, a 1-Ø bi-directional CLLC resonant converter with dc-bus voltage control and power reimbursement is developed and it is shown in Fig. 9. In both buck and boost mode, this converter can be controlled. The Critical considerations are the optimal design on the bi-directional full-bridge CLLC resonant converter, the ZVS function for the primary control on IGBTs, and the soft switching of the output rectifiers. Under mid and high load conditions, the bidirectional CLLC resonant converter have a good efficiency feature. These prototype converter overall power conversion efficiency is near to 97.8 percent at 4 kW. Because of the symmetrical circuit, it offers a certain voltage gain feature with bidirectional power flow, which minimizes the complexity in the control system and promotes power management. Besides, on both sides of the transformer, it distributes the resonant capacitors, which significantly decrease the voltage burden of the resonant capacitor compared to the LLC converter [76]. However, the CLLC converter has identical configuration to the LLC converter. Owing to its similarities with the LLC converter, the state of the ZVS and the lack of output voltage and power for high operating range. The consistency for the CLLC is another problem, because the voltage gain profile versus frequency tends to be constant in unique frequency intervals [77].

In [78], an auxiliary transformer is added to solve the above problems to assist realize full-bridge ZVS while enhancing power regulation. To achieve robust power control on high operating range, an absolute parameter design methodology is given in Ref. [79]. In Ref. [80], a design technique is presented that to controls the wide voltage gain necessities and integrated magnetic apparatuses are used to boost the power density. It is necessary to decrease the number of active devices in the topology at several cases. The half-bridge converters are one way of accomplishing these. Only four active switches are used by the half-bridge converters, which reduces the cost. The applied voltage is one-half of the dc-link voltage, compared with the full-bridge model. When the MV applications are to be used, this function is useful for the high-frequency transformer design. The current stress on the active machinery is doubled and the level of opportunity accessible to work the converter is diminished.

**Fig. 10:**
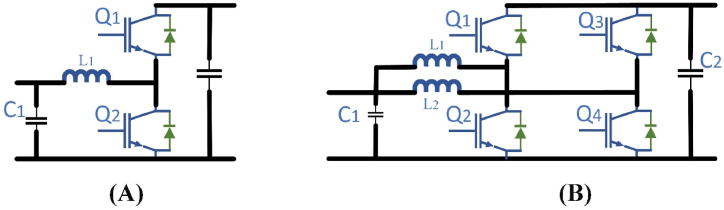
Non-isolated bi-directional dc-dc converter topologies (A) Half-bridge (B) Interleaved half-bridge.

Despite an isolated one, the non-isolated dc/dc converter can be utilized while generating the floating power supply into the vehicle battery if the charging device has configured, the advantage of isolation is to offered a distinct power conversion stage on EFC system. In general, it has advantages of simple design, high performance, less cost, reliability, and etc. There are a variety of non-isolated bi-directional DC-DC converters mentioned in the literature [[81], [82], [83], [84]]. These can be categorized into simple topologies, such as the half-bridge converter and the interleaved half-bridge converter, as shown in Fig. 10(A) and Fig.(B) respectively. Half-bridge is a commonly used model that works like buck or boost mode by using two active switches. Based on the half-bridge converter operation, it can possible to design the cascaded half-bridge and demonstrated interleaved half-bridge from the derived topologies. Due to synchronous rectification, the achievable performance of bidirectional converters is more compared to that of unidirectional. Second, with bidirectional operation the control does not involve difficulty in non-isolated dc/dc converters, unlike the isolated dc/dc converters.

**Fig. 11:**
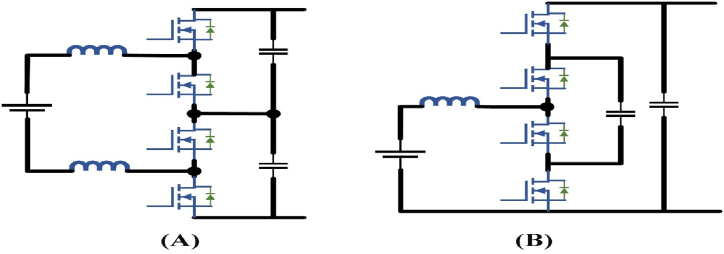
Dc fast chargers for Non-isolated dc-dc converters (A) bidirectional three-level boost (B) three-level flying capacitor.

In [85] bidirectional power flow is implemented to provide improved harmonic output compared to the boost converter. If the same inductor is used in the three-level boost converter the current ripples are very less approximately one-fourth of the boost converter, which ensures it is a smaller one that should be used in suitable for current ripple requirements. Therefore, the performance of the three-level boost converter is increased with large electromagnetic interference (EMI) and decreased the magnetic components size regarding common mode noise is described in Ref. [86]. In Refs. [87,88], the authors suggested the three-level boost converter for interfacing EV battery with a bipolar bus because of its three-level design, in an EV charging station topology as shown in Fig. 11(A). A flying capacitor converter is an another possible way of three-level topology for rapid chargers as shown in Fig. 11(B). Rather than a boost converter, the use of a smaller inductor is made possible by this three-level topology. Due to the flying capacitors existence, the short-circuit security design is too difficult. In addition, the flying capacitor converter switching commutation loop involves the upper and lowermost devices which are greater than that of the boost and three-level boost converter [89], which causes unwanted voltage overshoot during switching states. In this prototype, the battery voltage is raised thrice that of the traction inverter bus voltage during 55 kW power generation [90]. Over the entire power spectrum, the efficiency is approximately near to 97 percent.

### 4.3 Multi-port bidirectional three-phase AC-DC Converter

**Fig: 12:**
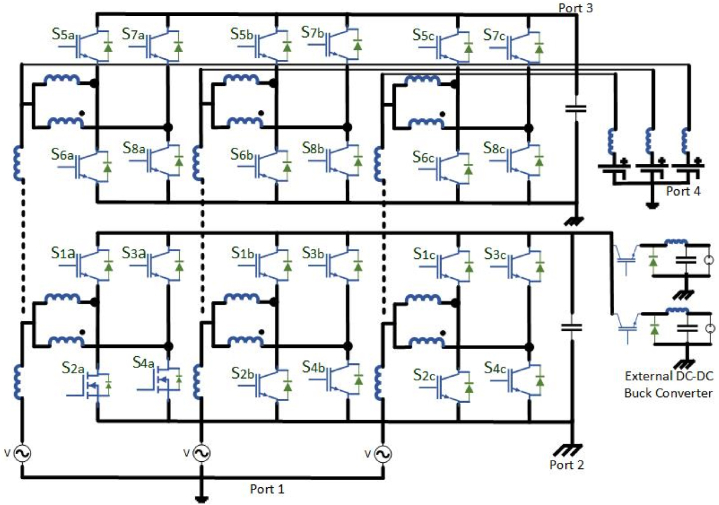
The configuration of the multi-port converter used in this proposal for the electric vehicle charging station (EVCS) microgrid.

The focus of the paper is on utilizing a multi-port bidirectional converter in the context of an electric vehicle charging station microgrid. This converter is a power electronic device capable of handling multiple power sources and loads, making it suitable for complex energy management scenarios. The primary advantage of using this multi-port converter in an electric vehicle charging station microgrid is its ability to integrate multiple power sources and loads into a single power conversion stage. This integration reduces the number of power conversion stages and devices required compared to other solutions. This can lead to increased efficiency and reduced complexity in the microgrid system. A photovoltaic (PV) system: Solar panels that generate electricity from sunlight. Battery Energy Storage System (BESS): Used for energy storage and management. Local grid: Likely the local electrical distribution network. Electric Vehicle Charging Station (EVCS): A facility for charging electric vehicles in Fig. 12. A 100 kW multi-port converter: This converter is a key component for power management and integration within the microgrid [91].

The effectiveness of the control scheme, especially during transients (temporary changes in power or voltage), confirms the feasibility of using this converter in electric vehicle charging station microgrid applications. This paper discusses the use of a multi-port bidirectional converter to streamline power management within an electric vehicle charging station microgrid. This approach aims to improve efficiency and reduce the complexity of the system while ensuring reliable operation and control during various power source conditions.

## 5 SST-BASED EFC architecture for MV

A balance among other variables like power density, modularity, and durability, it requires most acceptable topology. In urban areas, a transformer less topology is more appropriate for high power density with bounded space. On the other side, back-to-back ac-dc-dc charging stations are suitable for high flexibility and simpler power. Charging stations are more preferable choices in shopping malls or next to highways. The transformer is not suitable in near to the shopping malls and it stores the leakage inductance and leakage energy which must take some path to discharge and causes high voltage spike on switches on the current-fed hand during switching triggers. For MW loads, to integrate BES and RES in fast-charging station with suitable bi-directional power flow converter seems to be more important. Charging costs are directly proportional to its speed. It becomes very high importance on economic purpose, the advantage of fast Charging is it slowly overcomes the cost of the charging.

Large deployments of fast chargers around the world have been taken place in recent years. Since EVs would require more power from the grid and it supplies power for other loads also, so undesirable harmonics will be injected into the grid. SST-based power electronic interfaces can be used for the MV grid to reduce the requirement for the MV-to-LV transformer. Its contains a unique characteristics compared to the conventional line-frequency transformer like improved controllability, current limiting capabilities, and greater performance at light load conditions [92]. The three-stage dc bus is designed specifically to interface PVs, battery-powered storage systems, EVs, other dc sources and dc loads [93,94]. The single-stage SST is proposed in Ref. [95]. To implement the SST technology with current power systems allows the change of communication layers. The shortage of fast-acting safety devices such as circuit breakers (CBs) to operate against short-circuit faults, circuit overloads, and over voltages are other major challenges [96]. In Refs. [97,98], SST topologies implementations are summarized and compared. The overtime usage of the battery leads to deterioration of the batteries SOH. The lifespan of the battery can be directly affected by the electro-chemical reaction. During fast charging, the battery has higher temperature because it is subjected to high current and voltage, due to this the thermal degradation will increase [99]. Different magnetic core materials are mentioned in Table 10 for the high-frequency transformer (HFT) design. The best choice for HFT implementation would be an Amorphous material.

**Table 10:** Different magnetic core materials for HFT.

Parameters	Nanocrystalline	Amorphous	Ferrite
Operating Temperature (K)	393–453	393–423	373–423
Permeability	20-200 k	1-100 k	1.5–15 k
Operating frequency	Medium	Medium	High
Flux density (T)	1.1–1.3	0.8-1.5	0.3-0.5
Cost	High	Medium	Low
Loss (20 kHz,0.2T) (W/Kg)	4–8	5–7	15–20
Composition	FeCuNbSiB	(Co)x(SiB)y	MnZn

The SSTs are used an identical modules in the building blocks to achieve the desired power and voltage levels in general. These models are linked in series to enhance the capability of voltage blocking at the input side while the modules at the output side are connected parallel to provide a large current with appropriate low dc voltage in order to be associated with the MV grid. In Ref. [100], eight modules on the medium voltage (MV) side are linked in series to share a Voltage of 8-kV ac.

**Fig. 13:**
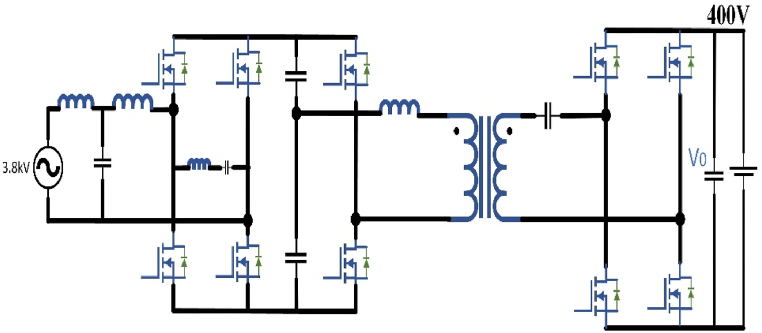
Medium Voltage interfaced single-module SST based dc fast charger.

Single module is developed for a 15 kW with an input source of 1 kV ac, it accomplished nearly 97.3 percentage. For the 3.8-kV MV ac input interface to the 400-V dc bus built on 10 kV SiC MOSFETs in the larger power-rated single-module SST is modeled and implemented in Fig. 13. The AC/DC front end has a full-bridge rectifier with a 7-kV internal bus. The switching frequency can be varied from 35 to 75 kHz with the Pulse Width Modulation (PWM) leg. For high-frequency transformer, at primary side LLC resonance is connected with the isolated dc-dc converter mode of a 13-kV SiC MOSFETs half-bridge. To reduce the switching losses in an ac-dc front end a unipolar modulation is implemented but the efficiency may reduce. The LLC stage works at a constant frequency and the ac-dc front end output voltage is controlled by tuning the internal bus voltage. At 25 kW power rating, it reaches 98 percent of the system performance was measured in Ref. [101].

Power electronic converters plays a major role by integrating of RES with EFC charging stations. So, these are directly connected to the MV grid as in the circumstance of the SST are prone to the applicable standards for security and power quality for MV apparatus, but by using transformer the cost and electromagnetic losses are increased. Besides, the non-isolated multiport converter excluded the transformer, since the size and cost are reduced, but very few switching losses are there, so the efficiency is increased. Besides, it does not suitable for high frequency applications. In this article wireless charging station techniques are not included. To improve the life cycle of fast-charging batteries future research is required.

### 5.1 Medium voltage multiport converter for an EV charging station

**Fig:14:**
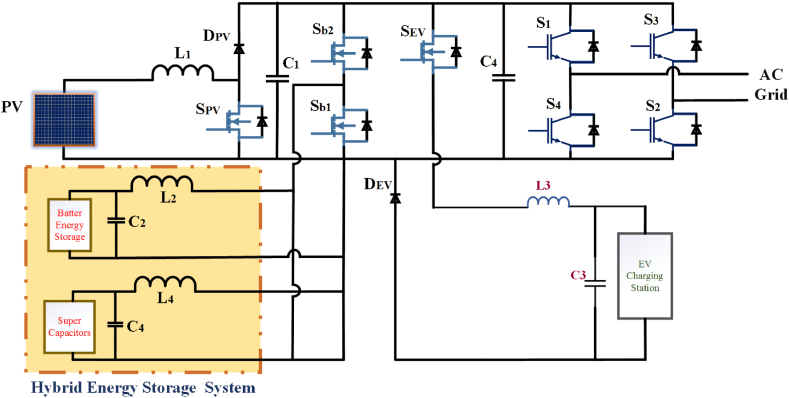
Medium Multiport converter for an EV charging station with the integration of RES.

It requires wide area distributed grid-connected charging stations due to the confined battery capacity and regular usage of EVs [102]. In specific EFC stations, the voltage sag, grid stability and reliability are affected at demand peak power overload and power gap problems [103]. To overcome the above issues SSTs has incorporated on-grid side. Only few researches suggested to integrate the PV generation with EV charging framework, although the integration of PV generation power remains a minor portion in charging stations. The EFC require large demand during day time, so the development of PV generation by using novel optimization techniques may ruins power depletion at peak hour loads. To maintain the reliability, power gap balance, and dc bus link voltage on RES systems the Hybrid Energy Storage System (HESS) has been incorporated. To control undesirable power ratings power electronic converters plays a major role. Instead of utilizing separate dc-dc converters, a multiport converter is implemented for EFC stations. Rather than using isolated, a non-isolated multiport converter can be designed from buck or boost converters having a characteristic of high power density, efficiency and compact design. The proposed Medium Multiport converter for an EV charging station with the integration of RES is shown in Fig. 14. The proposed system having a bidirectional power flow from HESSs to the grid. When the PV generation is surplus, the HESS has been utilized to sustain the constant dc-link voltage. If the PV generation is sufficient, the PV power can be delivered to the EFC stations. In an ideal case of the EFC station, the PV power can be delivered to the HESS. If battery SOC is full (∼100 %), then the PV generation can be delivered to the grid. Both the PV and HESS are in shortage of power generation, when the demand in EFC station in this condition the power can be delivered from the grid. The switching modes of operation mentioned in Table 11. Table 12 describes the comparison between Isolated SST-based and Non-isolated based multiport converter EFC station. Table 13 mentions the different medium voltage DC fast chargers for EV.

**Table 11:** Different switching modes for an EV Charging operation.

S_pv_	S_b1_	S_b2_	S_EV_	Power flow
Close	Open	Close	Open	Solar to HESS
Close	Open	Open	Open	Solar to Grid
Close	Open	Open	Close	Solar to EV
Open	Close	Open	Close	BES to EV
Open	Close/Open	Open/Close	Close	Grid to EV

**Table 12:** Comparison between Isolated SST-based and Non-Isolated Multiport converter EFC charging stations.

Parameters	Isolated SST-based Converter	Non-isolated Multiport Conveter
Safety and Protection	High	Moderate
Semiconductor failure	High	Low
Short citcuit or over voltage	Less	Moderate
Lighting surges	Less	High
EMI	Employed	Not Employed
Stability	High	Moderate
Cost	High	Very Low

**Table:13:** Different medium voltage DC fast chargers for EV.

Topologies	Diodes-Switches-Transformers In single unit	Design	Power flow	Control system	Switch Utilization	Efficiency	Cost
Ref. [86]	10-10-01	Modular	Unidirectional	Moderate	Good	High	Low
Ref. [90]	10-08-01	Modular	Bidirectional	Complex	Very good	High	Moderate
Ref. [107]	06-12-01	Modular	Bidirectional	Advanced	Good	High	Moderate
Ref. [108]	03-08-01	Modular	Bidirectional	Complex	Average	High	Low
Ref. [109]	08-04-02	Modular	Bidirectional	Moderate	Good	High	Moderate
Ref. [110]	00-12-01	Modular	Bidirectional	Complex	Good	High	Low

### 5.2 Multiport dual active bridge (DAB)

**Fig:15:**
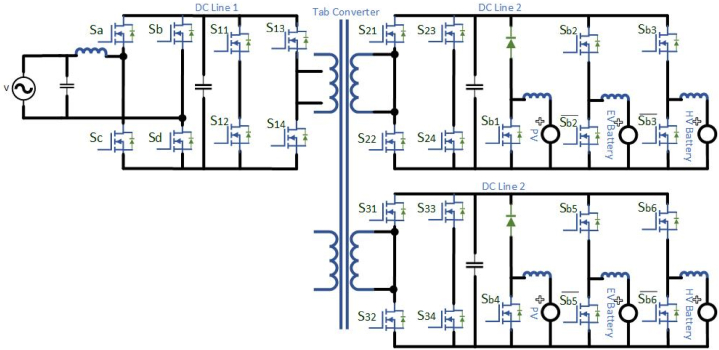
A variety of converters, including multiport dual active bridge (DAB) and triple active bridge (TAB) converters, bidirectional DC-DC converters, and unidirectional DC-DC converters.

The DAB converter is a multi-port power electronic converter topology used for high-frequency AC to DC or DC to DC power conversion. It typically consists of two active bridges connected through an isolation transformer. DAB converters are known for their ability to provide multiple ports for power transfer, making them suitable for applications requiring energy flow between multiple sources or loads. This topology approach, when combined with a multi-winding transformer, enables the integration and control of multiple DC voltage sources. This can be particularly useful in applications where there are multiple energy sources or loads that need to be interconnected and managed efficiently, such as microgrids, renewable energy systems, or hybrid power systems. This architecture represents an advanced and versatile solution for EV charging stations that aims to optimize energy utilization, support renewable energy integration, and ensure grid compliance through electrical isolation in Fig. 15. It reflects the growing importance of smart and sustainable charging infrastructure in the electric vehicle ecosystem [104].

### 5.3 Isolated three-port DC/DC Converters for Hybrid Charging Stations

**Fig:16:**
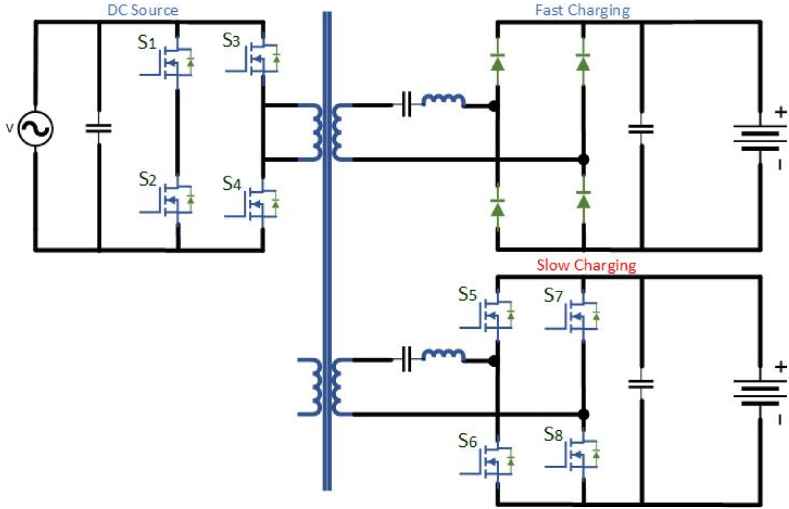
Three-port isolated DC/DC converters designed for hybrid charging stations.

The proposed three-port converter offers notable advantages, primarily stemming from its streamlined structure with fewer components. This design choice results in cost reduction and a smaller overall system footprint when compared to the use of separate charger systems. A straightforward control method, employing phase shift and frequency modulations, has been developed to effectively manage the output power of both the fast and slow charging ports simultaneously. Furthermore, an optimal phase shift angle has been determined to minimize transformer current when the DAB converter operates exclusively for slow charging purposes. Fig. 16 represents the design of isolated Three-Port DC/DC Converters for Hybrid Charging Stations.

Various isolated DC/DC converter topologies, including the phase-shift full-bridge converter, resonant converter, and DAB converter, can be employed for DC fast chargers. To validate the converter's performance, a 5-kW SiC-based prototype was successfully assembled, an impressive power density of 2.74 kW/dm3 was attained, thanks to the utilization of a high switching frequency that reached up to 220 kHz. Furthermore, the experimental measurements yielded the highest efficiency, reaching an impressive 98.2 % [105].

### 5.4 Three port DC-DC-AC Converter

**Fig:17:**
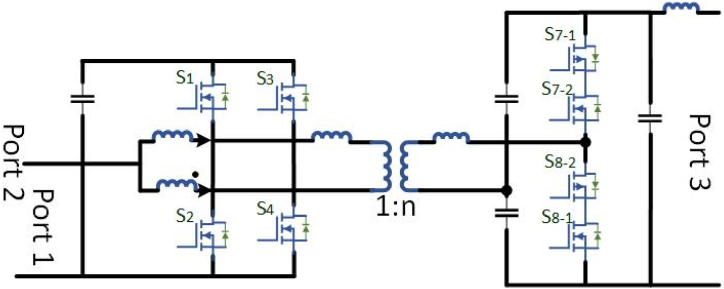
The Two-Port Converter (TPC) utilizes a dual active bridge topology.

This converter is based on a dual active bridge topology. In this design, the secondary bridge consists of four switches that can operate bidirectionally, allowing it to connect directly to an AC port. The primary bridge can also function as an interleaved bidirectional boost converter by connecting two inductors across the transformer primary, creating an additional input DC port. This converter enables fully soft-switched three-port power conversion with fewer active devices compared to existing designs in Fig. 17. It employs two interleaved inductors, L3 and L4, which have larger values compared to the leakage inductances, L1 and L2. These leakage inductances are found on both the primary and secondary sides of the high-frequency transformer [106].

For simplification, the analysis considers the lumping of leakage inductances onto either the primary or secondary side. With pulse frequency modulation, the converter ensures zero voltage switching (ZVS) for all power levels, achieving a peak efficiency of 96.5 %.

## 6 Insights and future development forecast for EFC with multiport converter of EV’s

### 6.1 Insights

A. **Increased EV Adoption:** The adoption of electric vehicles has been steadily increasing due to environmental concerns and advancements in EV technology. This has created a growing demand for fast and convenient charging solutions.
B. **Integration of RES:** The integration of renewable energy sources, such as solar and wind, into charging stations is gaining importance. This not only reduces the carbon footprint of EV charging but also makes use of clean energy sources.
C. **Energy Storage:** To ensure a continuous power supply, energy storage solutions like batteries are often integrated into charging stations. These batteries can store
D. excess energy from renewable sources and provide a stable power supply even when renewable generation is low.
E. **Multiport Converters:** Multiport converters play a crucial role in efficiently managing the power flow between the grid, renewable energy sources, energy storage systems, and multiple EVs. These converters help balance energy distribution and ensure fast and reliable charging.
F. **Grid Integration:** Charging stations need to be integrated into the grid effectively to avoid overloading and to optimize energy usage. Advanced grid management systems and smart charging technologies are essential for this purpose.

### 6.2 Future development forecast

A. **Ultra-Fast Charging:** Charging stations will continue to evolve, offering even faster charging speeds. This will require advancements in battery technology and power electronics to deliver higher currents safely.
B. **Enhanced Energy Efficiency:** Research and development will focus on improving the efficiency of energy conversion and storage systems. This will reduce energy losses during charging, making the process more environmentally friendly.
C. **Grid Resilience:** Charging stations will become an integral part of grid resilience efforts. They will provide grid support during peak demand periods and have the ability to disconnect from the grid during emergencies, relying on stored energy and renewables.
D. **Vehicle-to-Grid (V2G) Integration:** V2G technology will become more widespread, allowing EVs to not only charge from the grid but also feed excess energy back into the grid when needed. This will further stabilize the grid and provide additional revenue streams for EV owners.
E. **Standardization:** As the EV charging infrastructure continues to expand, there will be a push for global standards in terms of connectors, communication protocols, and safety features. This will ensure interoperability and ease of use for EV owners.
F. **Increased Use of Artificial & intelligence (AI) and Machine Learning:** AI and data analytics will play a significant role in optimizing charging station operations. These technologies will help predict demand, manage energy flow, and offer personalized charging experiences.
G. **Environmental Sustainability:** There will be a greater emphasis on making charging stations environmentally sustainable. This includes using recycled materials in construction, minimizing energy consumption, and implementing eco-friendly designs.
H. **Public and Private Partnerships:** Collaboration between governments, utility companies, automakers, and private businesses will be crucial to fund and deploy a comprehensive network of charging stations. Incentives and subsidies may also play a role in accelerating development.

Renewable energy from distributed sources is becoming more common in regular power grids. However, it can cause issues with backup protection systems, making them less effective or even useless in some situations. This paper suggests a new way to solve this problem by simulating a fault current artificially. There are two methods proposed. First, an extra current transformer is added to the protection system, and its output is adjusted based on how serious the protection system's issue is. This adjusted output is then combined with the main current transformer's output to improve sensitivity. Second, a computer-based protection system is used, and its settings are changed by introducing a new variable called the "artificial fault current factor" to better handle fault currents [111].

In the study of integrated energy systems (IES) using a lot of renewable energy, power-to-gas (PtG) is seen as a crucial tool for managing peak energy demand due to its ability to convert and store energy. To make our system operations more precise and economically sound, we've improved the PtG model by including the use of waste heat from chemical reactions. With this in mind, we're exploring the idea of creating an energy hub that connects electricity, gas, and heat around the PtG process. We've also introduced a shared heat storage system (SHSS) to enable energy sharing between different energy networks and the SHSS [[112], [113], [114]]. Table 14 mentions a summary of currently available various multiport electric vehicle (EV) chargers [115]. Further research is needed to integrate these converter technologies with emerging smart grid and IoT frameworks to enhance their functionality and interoperability. Practical implementation and scalability of these advanced converters in real-world charging stations remain an area requiring more research and development.

**Table 14:** A summary of presently available multiport electric vehicle (EV) chargers is as follows.

Ref.	Quantity of Ports and Interface Configuration	Topology	Power (kW)	Modes of power flow/Peak Efficiency	Merits	Demerits
[91]	4 Ports: AC Grid, HESS, PV &EV	Full bridge (AC Grid), Buck at EV side.	100	PV→ EV, PV→ Grid, PV→ ES, ES→ Grid, ES→ EV, Grid→ ES	It is suitable for high power and ultra-fast charging, Low THD, High Power factor, Simple & effective control.	No V2G Operation, reliability issue due to series connection between EV & Energy storage.
[104]	4 Ports: AC Grid, HESS, PV & EV	Triple Active Bridge, PV (DC-DC Boost), Bidirectional DC-DC (HESS, EV)	12	PV→ HESS, PV→ AC Grid, PV → EV, Grid→ HESS, HESS→ EV, HESS→ Micro Grid,	Electrical Isolation, V2G Operation, Soft Switching, Support wide Variety of dc Sources through multi-winding transformer.	Hard switching, Transformer design is challenging for high power flow, control analysis is complex.
[105]	3 Ports: DC source, AC-DC rectifier, EV, EV/HESS	Triple Active Bridge, Dual Active Bridge, Series Resonant	5	Grid→EV, Grid→ HESS, HESS→Grid	Multiple EV Charging, Low cost, High Efficiency	RES is not considered, Transformer design is complex.
[106]	3 Ports: AC Grid, PV, EV	Dual Active Bridge, Interleaved Boost	0.2	Grid→EV EV→ Grid, PV→ EV, PV→ Grid.	Power density is high, soft switching, simple control	Large output filter is required to maintain low THD

## 7 Conclusion

The main conclusion of the article is that integrating advanced control algorithms, efficient MPPT techniques, and multiport converter technology in electric vehicle (EV) charging stations, particularly those utilizing renewable energy sources like solar power, can significantly enhance their efficiency, reliability, and sustainability. These technological advancements enable optimal power extraction, improve power quality, support grid stability, and facilitate better load management. However, challenges remain, such as the need for more robust control algorithms to handle adverse grid conditions, cybersecurity measures, and simplifying complex implementations. The future of EV charging infrastructure looks promising, focusing on scalability, efficiency, sustainability, policy support, and market demand.

## Funding statement

No funding was supported for this research work.

## Data availability statement

No data available to this article.

## CRediT authorship contribution statement

**Jayaprakash Suvvala:** Writing – original draft, Validation, Software, Methodology, Investigation, Formal analysis, Data curation, Conceptualization. **Sathish Kumar K:** Writing – review & editing, Writing – original draft, Visualization, Validation, Supervision, Software, Formal analysis. **C. Dhananjayulu:** Writing – review & editing, Supervision, Resources, Project administration, Data curation. **Hossam Kotb:** Writing – review & editing, Visualization, Methodology, Investigation, Conceptualization. **Ali Elrashidi:** Writing – review & editing, Writing – original draft, Visualization, Resources, Funding acquisition.

## Declaration of competing interest

The authors declare that they have no known competing financial interests or personal relationships that could have appeared to influence the work reported in this paper.
